# High visceral fat-to-muscle ratio is an independent factor that predicts worse overall survival in patients with primary epithelial ovarian, fallopian tube, and peritoneal cancer

**DOI:** 10.1186/s13048-023-01098-1

**Published:** 2023-01-21

**Authors:** Sooji Ham, Jin Hwa Choi, Soo Gui Shin, Eun-Ju Lee

**Affiliations:** 1 Department of Obstetrics and Gynecology, Chung-Ang University School of Medicine, Chung-Ang University Hospital, 102, Heuksuk-Ro, Dongjak-Gu, Seoul, 06973 Korea; 2 Department of Radiation Oncology, Chung-Ang University School of Medicine, Chung-Ang University Hospital, Seoul, Korea; 3 Department of Microbiology & Molecular Genetics, University of California, Irvine, CA 92697 USA

**Keywords:** Visceral fat-to-muscle ratio, Ovarian cancer, Overall survival, Thrombocytosis, Sarcopenia

## Abstract

**Background:**

The intra-abdominal cavity, surrounded by adipocytes, is the main metastatic site of epithelial ovarian, fallopian tube, and peritoneal cancer. Epidemiological and molecular studies have demonstrated a link between adipose tissue and ovarian cancer. However, the clinical significance of fatty tissue has not been elucidated. Thus, we investigated the clinical significance of body composition in patients with epithelial ovarian, fallopian tube, and peritoneal cancer.

**Methods:**

Fat and skeletal muscle areas were measured using software based on pretreatment computed tomography scans at the third lumbar vertebra. Fat-to-muscle ratios were calculated using the total (visceral and subcutaneous) fat area or visceral fat area. High fat-to-muscle ratios were defined by values greater than the mean. Sarcopenia was defined as a skeletal muscle index < 38.7 cm^2^/m^2^. The clinicopathological parameters and survival of 153 patients were analyzed.

**Results:**

High visceral fat-to-muscle ratios and sarcopenia at the time of diagnosis were observed in 43.8% and 33.3% of the patients, respectively. Multivariate analysis showed that high visceral fat-to-muscle ratio (*p* = 0.014), advanced Federation of Gynecology and Obstetrics stage (*p* = 0.008), and chemoresistance (*p* = 0.027) were independent factors for worse overall survival. Patients with high visceral fat-to-muscle ratios were older, had higher body mass indexes, and were more likely to have diabetes/hypertension, serous cancer subtypes, and implementation of neoadjuvant chemotherapy than those with low visceral fat-to-muscle ratios. The platelet count was significantly higher in the high visceral fat-to-muscle ratio group than in the low visceral fat-to-muscle ratio group (*p* = 0.011).

**Conclusions:**

Pretreatment visceral fat area could be an independent predictive factor of overall survival in patients with epithelial ovarian, fallopian tube, and peritoneal cancer and may be significantly associated with thrombocytosis.

**Supplementary Information:**

The online version contains supplementary material available at 10.1186/s13048-023-01098-1.

## Background

Epithelial ovarian cancer is one of the most lethal gynecologic malignancies, and its incidence in Korea is gradually increasing [1]. Approximately 70% of patients are found to have advanced disease status at the time of diagnosis. Despite extensive surgery and chemotherapy, most patients experience disease recurrence in advanced cases [2, 3]. Attempts to improve survival have been made, and the modification of patient factors, including body composition, is essential for adequate treatment. Therefore, the identification of patient factors that affect survival is required.

Ovarian cancer is known to primarily spread into the abdominal cavity, which is largely surrounded by visceral fatty tissue, and rarely metastasizes outside the abdominal cavity. Compared to subcutaneous fatty tissue, visceral fatty tissue is metabolically active, producing hormones and inflammatory cytokines that are involved in tumor progression [4]. Moreover, intraabdominal adipocytes promote ovarian cancer metastasis by providing an energy source and adipokines [5]. Therefore, visceral fatty tissue may play an essential role in ovarian cancer progression.

Body composition, such as fat, muscle, and bone, is closely related to pathological conditions as well as normal physiological states [6]. The clinical significance of body composition has been studied in malignancy, and sarcopenia, the most common abnormality in body composition, has been identified as a prognostic factor for various malignancies. However, in ovarian cancer, sarcopenia is not associated with prognosis [7–9]. Instead, a high volume of fat tissue was correlated with an increased risk of 13 distinct types of cancer, including ovarian cancer, in an epidemiologic study [10], and visceral obesity in patients with advanced ovarian cancer was reported to be associated with a higher occurrence of postoperative complications [11]. However, the clinical significance of visceral fat in ovarian cancer has not been elucidated.

In this study, we aimed to investigate body composition, including visceral fat, subcutaneous fat, and skeletal muscle mass, at the time of diagnosis, and its association with prognosis in patients with epithelial ovarian, fallopian tube, and peritoneal cancer (EOFPC).

## Results

### Patient characteristics

The parameters of body composition at diagnosis and the clinicopathological characteristics are summarized in Table 1. The mean skeletal muscle index (SMI) and visceral (vFMR) and total fat-to-muscle ratio (tFMR) values were 41.53, 0.83, and 2.22, respectively. The majority of patients were under 60 years old (60.1%), had a body mass index (BMI) lower than 25 kg/m^2^ (66.7%), had serous-type carcinoma (65.4%), and underwent primary debulking surgery as a first-line therapy (88.2%). The median follow-up time after finishing the first-line treatment was 42.7 months (range, 0–150 months). Disease recurrence occurred in 65 patients (42.5%), and 32 patients died. Sarcopenia at diagnosis was observed in 51 patients (33.3%).

**Table 1:** Body composition parameters at diagnosis and clinicopathological characteristics of 153 patients

	Mean ± standard deviation (range)	Number (%)
Age at diagnosis, year	55.83 ± 13.17	
BMI, kg/m^2^	23.57 ± 4.21	
Visceral fat area, cm^2^	85.52 ± 67.53 (6.36 - 407.00)	
Subcutaneous fat area, cm^2^	141.85 ± 64.71 (27.10 - 356.00)	
Total fat area, cm^2^	227.37 ± 122.64 (27.10 - 356.00)	
Skeletal muscle area, cm^2^	101.99 ± 16.35 (66.00 - 162.00)	
Skeletal muscle attenuation	42.42 ± 9.27 (15.30 - 59.00)	
Skeletal muscle index (SMI), cm^2^/m^2^	41.53 ± 6.20 (2711.00 - 64.00)	
Visceral FMR	0.83 ± 0.59 (0.08 - 3.23)	
Total FMR	2.22 ± 1.08 (0.41 - 6.74)	
Age at diagnosis		
<60		92 (60.1)
≥60		61 (39.9)
DM/HTN		
No		105 (68.6)
DM or HTN		48 (31.4)
Menopause		
No		55 (35.9)
Yes		98 (64.1)
BMI		
<25		102 (66.7)
≥25		51 (33.3)
Cell type		
Serous		100 (65.4)
Non-serous		53 (34.6)
First-line therapy strategy		
PDS		135 (88.2)
NAC		18 (11.8)
Surgical optimality		
Optimal		115 (75.2)
Suboptimal		38 (24.8)
FIGO stage		
1		49 (32.0)
2		8 (5.2)
3		71 (46.4)
4		25 (16.4)
Chemoresponse		
Sensitive		112 (73.2)
Resistant		20 (13.1)
Unknown		7 (4.6)
Recur		
No		75 (49.0)
Yes		65 (42.5)
Unknown		13 (8.5)
SMI		
<38.7		51 (33.3)
≥38.7		102 (66.7)

*BMI* Body mass index, *FMR* Fat -to-muscle ratio, *DM/HTN* Diabetes/hypertension, *PDS* Primary debulking surgery, *NAC* Neoadjuvant chemotherapy

### Higher visceral fat is an independent factor that predicts worse overall survival

**Fig. 1:**
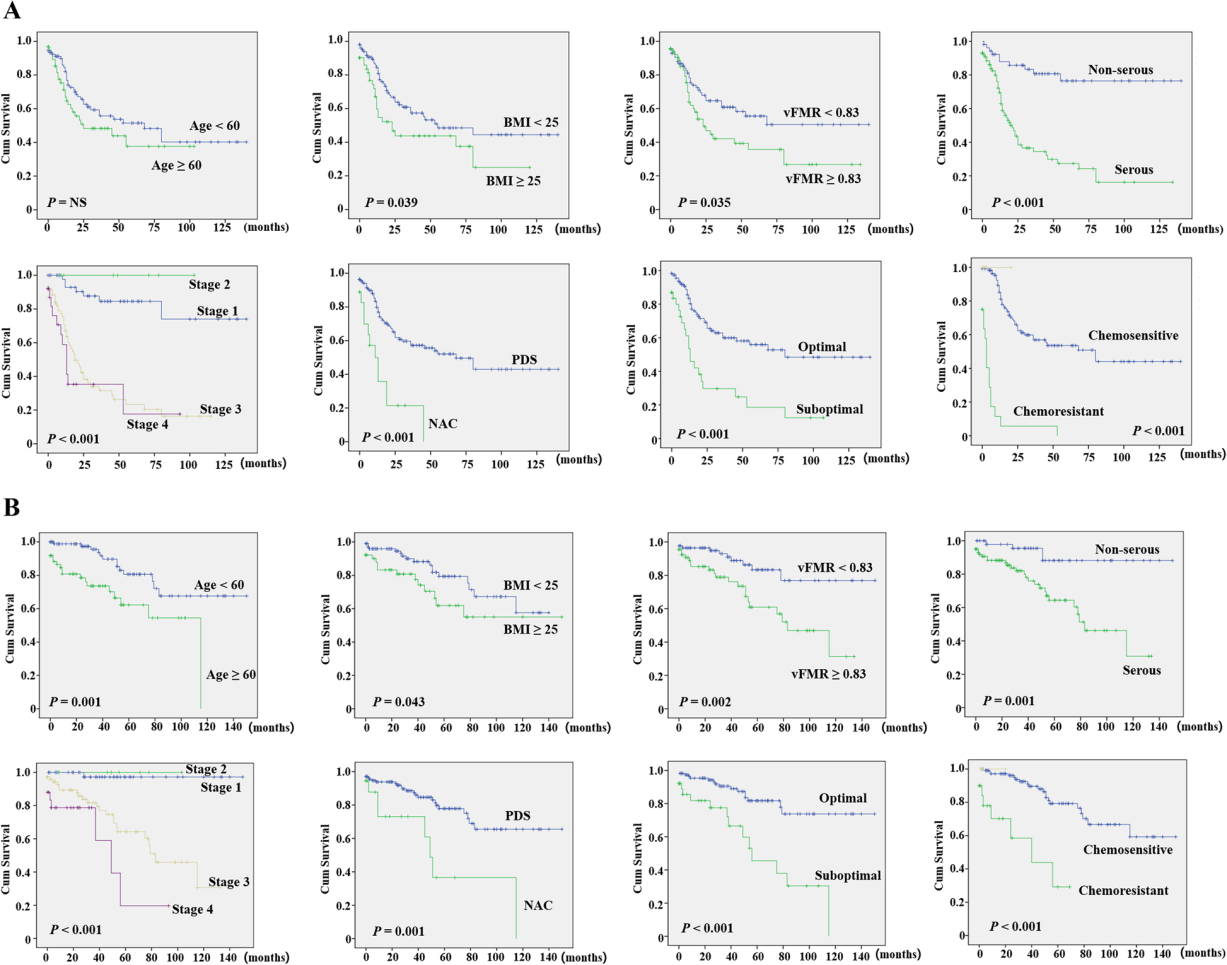
Kaplan–Meier survival curves and log-rank tests. Disease-free survival (**A**) and overall survival (**B**) values are shown

The present study analyzed body composition and its relationship with the prognosis of patients with primary EOFPC. Univariate analysis showed that a higher BMI, serous subtype, implementation of neoadjuvant chemotherapy (NAC), higher Federation of Gynecology and Obstetrics (FIGO) stage, suboptimality, chemoresistance, and high vFMR were significantly associated with worse disease-free survival (DFS) (Table 2 and Fig. 1A). Multivariate analysis showed that higher BMI, serous subtype, implementation of NAC, higher FIGO stage, and chemoresistance were independent factors for DFS. In addition, older age, higher BMI, serous subtype, implementation of NAC, suboptimality, higher FIGO stage, chemoresistance, and high vFMR were significantly associated with worse overall survival (OS) in the univariate analysis (Table 2 and Fig. 1B). Multivariate analysis revealed that higher FIGO stage, chemoresistance, and high vFMR were independent factors for worse OS. In contrast to vFMR, tFMR and SMI were not significantly associated with survival.

**Table 2:** Survival analysis

		Disease-free survival				Overall survival			
		Univariate	Multivariate			Univariate	Multivariate		
	N (%)	*p*-value	*p*-value	HR	95% CI	*p*-value	*p*-value	HR	95% CI
Age at diagnosis									
< 60	92 (60.1)	0.184	-			0.001	0.483	1.381	0.559–3.411
≥ 60	61 (39.9)								
DM/HTN									
None	105 (68.6)	0.455	-			0.115	_		
DM or HTN	48 (31.4)								
Menopause									
no	55 (35.9)	0.437	-			0.231	_		
yes	98 (64.1)								
BMI									
< 25	102 (66.7)	0.039	0.020	1.434	1.058–1.945	0.043	_		
≥ 25	51 (33.3)								
Histologic type									
Serous	100 (65.4)	< 0.001	-			0.029	_		
Endometroid	12 (7.8)								
Clear cell	14 (9.2)								
Mucinous	20 (13.1)								
Transitional cell	3 (1.9)								
MMMT	4 (2.6)								
Cell type									
Serous	100 (65.4)	< 0.001	0.017	2.530	1.178–5.433	0.001	0.716	1.238	0.390–3.926
Non-serous	53 (34.5)								
First-line therapy									
PDS	135 (88.2)	< 0.001	0.049	2.017	1.000–4.068	0.001	0.967	1.023	0.342–3.052
NAC	18 (11.8)								
Surgical optimality									
Optimal	115 (75.2)	< 0.001	0.377	1.328	0.706–2.499	< 0.001	0.295	1.573	0.673–3.676
Suboptimal	38 (24.8)								
FIGO stage									
1	49 (32.0)	< 0.001	0.009	1.571	1.116–2.210	< 0.001	0.008	2.516	1.260–5.024
2	8 (5.2)								
3	71 (46.4)								
4	25 (16.4)								
Chemo-response									
Sensitive	112 (73.2)	< 0.001	0.012	2.007	1.160–3.475	< 0.001	0.027	2.786	1.121–6.923
Resistant	20 (13.1)								
Unknown	21 (13.7)								
tFMR									
< 2.22	79 (51.6)	0.328	-			0.303	_		
≥ 2.22	74 (48.4)								
vFMR									
< 0.83	86 (56.2)	0.035	0.989	0.995	0.547–1.812	0.002	0.014	3.302	1.269–8.594
≥ 0.83	67 (42.8)								
SMI									
< 38.7	51 (33.3)	0.583				0.051	_		
≥ 38.7	102 (66.7)								

*DM/HTN* Diabetes/hypertension, *BMI* Body mass index, *PDS* Primary debulking surgery, *NAC* Neoadjuvant chemotherapy, *FMR* Fat-to-muscle ratio, *SMI* Skeletal muscle index

### Comparison between the low and high vFMR groups

To assess the characteristics of patients with high vFMR, we subdivided the patients into low and high vFMR groups according to the mean value (0.83) and compared the body composition, laboratory data, and clinicopathological parameters between the two groups (Table 3). In the high vFMR group, age, BMI, and subcutaneous fat levels were significantly higher than in the low vFMR group, whereas skeletal muscle area (SMA) and SMI did not differ between the two groups. Skeletal muscle attenuation was significantly lower in the high vFMR group than in the low vFMR group owing to the high lipid content in the muscles.

**Table 3:** Comparison of parameters of patients with low and high vFMR

	Low vFMR (*n* = 86)	High vFMR (*n* = 67)	*p*-value
Age at diagnosis, year	51.97 ± 13.40	60.79 ± 11.20	< 0.001^*^
BMI, kg/m^2^	21.99 ± 2.81	25.62 ± 4.82	< 0.001^*^
Visceral fat area, cm^2^	42.81 ± 21.38	140.33 ± 66.99	< 0.001^*^
Subcutaneous fat area, cm^2^	114.08 ± 49.84	177.50 ± 64.39	< 0.001^*^
SMA, cm^2^	101.36 ± 15.85	102.81 ± 17.06	0.588^*^
Skeletal muscle attenuation	45.33 ± 8.58	39.19 ± 8.99	< 0.001^*^
Skeletal muscle index	40.78 ± 6.26	42.48 ± 6.03	0.092^*^
WBC, /μL	6,616.40 ± 1,790.30	7,338.70 ± 2,596.49	0.055^*^
Absolute neutrophil count, /μL	4,331.50 ± 1,641.54	5,016.50 ± 2,496.38	0.056^*^
Lymphocyte, /μL	1,674.40 ± 661.43	1,686.50 ± 650.75	0.911^*^
Neutrophil to lymphocyte ratio	3.15 ± 2.27	3.66 ± 3.31	0.265^*^
Hemoglobin, g/dL	11.93 ± 1.62	12.14 ± 1.52	0.392^*^
Platelet, 10^3^/μL	288.33 ± 96.23	334.39 ± 117.89	0.011^*^
Albumin, g/dL	3.94 ± 0.53	3.94 ± 0.39	0.980^*^
PNI	49.11 ± 6.07	47.70 ± 6.55	0.179^*^
Age			
< 60	63	29	< 0.001^†^
≥ 60	23	38	
DM/HTN			
No	70	35	< 0.001^†^
DM or HTN	16	32	
Menopause			
No	40	15	0.002^†^
yes	46	52	
BMI			
< 25	71	31	< 0.001^†^
≥ 25	15	36	
Cell type			
serous	48	52	0.005^†^
non-serous	38	15	
First-line therapy strategy			
PDS	80	55	0.037^†^
NAC	6	12	
Optimal			
Optimal	65	50	0.892^†^
suboptimal	22	16	
FIGO stage			
1	37	12	NS^††^
2	3	5	
3	32	39	
4	14	11	
Chemoresponse			
sensitive	61	51	0.896^†^
resistant	12	8	
unknown	4	3	
Recur			
No	48	27	0.15^†^
recur	31	34	
F/U loss	7	6	
SMI			
< 38.7	34	17	0.065^†^
≥ 38.7	52	50	

*BMI* Body mass index, *SMA* Skeletal muscle area, *WBC* White blood cell, *PNI* Prognostic nutritional index, *DM/HTN* Diabetes/hypertension, *PDS* Primary debulking surgery, *NAC* Neoadjuvant chemotherapy, *SMI* Skeletal muscle index

*Student’s *t*-test; † Chi-squared test; †† Fisher’s exact test

The comparison of the laboratory results at diagnosis showed that the mean platelet count was significantly higher in the high vFMR group than in the low vFMR group, while there were no differences in white blood cell (WBC) count, neutrophil or lymphocyte counts, hemoglobin, albumin, or prognostic nutrition index (PNI). We next compared the frequency of thrombocytosis, defined as > 400x10^3^/μL, between the two groups. The results showed that thrombocytosis was significantly more frequent in patients in the high vFMR group (17 of 67, 25.4%), than in patients in the low vFMR group (9 of 86, 10.5%) (*p* < 0.02).

In the comparison of clinicopathological parameters, patients with high vFMR were older and more frequently had diabetes/hypertension (DM/HTN) or were menopausal than those with low vFMR. In addition, the presence of serous subtype and implementation of NAC were significantly associated with high vFMR.

## Discussion

This study demonstrated that visceral fat at EOFPC diagnosis could be an independent prognostic factor by showing a significant association between vFMR and OS. Subcutaneous fat, total fat, and sarcopenia were not significant factors in the current analysis. Patients with a vFMR higher than the average value of this cohort were older, had a higher BMI, and more frequently had DM/HTN, serous subtype, and implementation of NAC compared to those with lower vFMR. These associated factors were unfavorable to the prognosis of patients, contributing to the significant association between high vFMR and low OS.

Three similar studies on the correlation between fat area and prognosis in epithelial ovarian cancer have been conducted. One report showed that higher tFMR was significantly associated with worse OS in patients with sarcopenia, but not in all patients; however, visceral fat was not analyzed, and only the serous ovarian subtype was included [7]. Two other reports analyzed the clinical significance of total fat area and showed no correlation with survival [12, 13]. In contrast to the visceral fat area at the level of the third lumbar vertebra (L3) on CT scans that was used in our study, one of the three reports measured the whole abdominal visceral fat area; however, it showed no clinical significance [13]. Consistent with previous reports, we did not find any clinical significance of the total fat area. Instead, we demonstrated that the visceral fat area at the L3 level on CT was a more reliable factor for predicting the survival of patients with EOFPC. Because previous reports did not analyze the visceral fat area at the L3 level on CT, this is the first report to demonstrate a significant association between the visceral fat area at the L3 level on CT and the prognosis of patients with EOFPC.

The clinical significance of visceral fat area depends on the type of malignancy. While higher visceral fat was significantly associated with better OS in gastric cancer [14], a higher visceral to subcutaneous fat ratio was significantly associated with worse OS in uterine cervical, pancreatic, and metastatic colorectal cancer [15–17]. Here, we provide additional evidence for ovarian cancer, supporting the significant association between a higher visceral fat area and worse OS.

There is evidence of a link between fat tissue and ovarian cancer. An epidemiological study showed that a high volume of fat tissue is correlated with an increased risk of 13 distinct types of cancer, including ovarian cancer [10]. The direct dissemination sites of epithelial ovarian cancer are the omentum, mesentery, and peritoneum, which are rich in adipocytes [18]. Molecular studies have demonstrated that epithelial ovarian cancer cells lead to phenotypic and metabolic alterations in adipocytes at the intraabdominal metastasis site and fatty acid production from adipocytes, resulting in epithelial ovarian cancer progression [5]. In addition, adipocytes are responsible for epithelial ovarian cancer treatment resistance through various mechanisms [19]. For example, adipocytes modulate survival genes by secreting leptin, interleukin (IL)-6, and IL-8, which activate the AKT and ERK survival pathways, and contribute to Taxol resistance [20, 21]. In addition, adipocyte-induced autophagy and adipocyte-derived mesenchymal stem cells also contribute to drug resistance [22, 23]. Taken together, visceral fat tissue may play an important role in epithelial ovarian cancer progression, and this is supported by our finding that high vFMR is an unfavorable prognostic factor in patients with EOFPC.

In this study, sarcopenia was not clinically significant. Sarcopenia is known to influence the survival of patients with various malignancies, such as gastric cancer [24], and breast cancer [25]. However, the results for ovarian cancer remain controversial. Most reports, including the current study, showed no association between sarcopenia and survival in patients with ovarian cancer [12, 26–32], whereas several studies reported a significant association [12, 32, 33]. Although a recent meta-analysis showed a significant correlation between sarcopenia and ovarian cancer survival, the authors carefully added that the source data were mainly retrospective in nature and of low quality [9]. Overall, further large-scale prospective studies with multiple centers are necessary to elucidate the clinical role of sarcopenia in patients with ovarian cancer.

Interestingly, the present study showed that platelet count was significantly higher and thrombocytosis was significantly more frequent in the high vFMR group than in the low vFMR group. The direct relationship between thrombocytosis and visceral fat area has never been assessed in population studies. Thrombocytosis is a paraneoplastic syndrome of ovarian cancer, and the mechanism is mainly explained by the higher plasma levels of thrombopoietin and IL-6 in patients with thrombocytosis compared to those without thrombocytosis [34]. In addition, platelet counts have been found to be higher in obese individuals than in non-obese controls [35]. This correlation is further supported by the observed reductions in platelet counts in the setting of weight loss following bariatric surgery [36]. Moreover, the secretion of thrombopoietin and IL-6 is enhanced in visceral adipose tissue, such as the omentum [21, 37]. Taken together, visceral adipose tissue may be an additional source of thrombopoietin and IL-6, which may explain thrombocytosis in patients with high vFMR; however, further studies are needed to prove this.

This study is the first to demonstrate the clinical significance of visceral fat in patients with EOFPC, whereas the previous studies that assessed the role of body composition did not focus on this factor. With this evidence, the role of visceral fat in ovarian carcinogenesis should be further addressed to achieve a better understanding of the cancer microenvironment, to evaluate the necessity of debulking visceral fat, and to integrate these findings into the prevention spectrum. Despite the present study’s important findings, it was limited by its retrospective nature and its small sample size from a single institution. Thus, further well-designed, prospective studies are required.

## Conclusions

This study showed a novel finding that high vFMR, but not sarcopenia, was an independent predictor of worse OS and associated with older age, higher BMI, the implementation of NAC, and the presence of DM/HTN, serous subtype, and thrombocytosis. Therefore, high vFMR could be a reliable patient factor affecting survival, and its modification should be considered in patients with EOFPC.

## Methods

### Patients

**Fig. 2:**
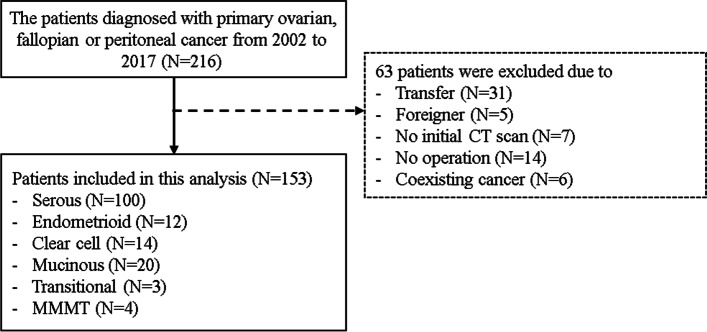
Flow diagram depicting patient selection process

A total of 216 patients diagnosed with primary EOFPC at Chung-Ang University Hospital between 2002 and 2017 were retrospectively identified. Of these, 63 were excluded from the study. Thirty-one patients were transferred immediately after diagnosis (N = 25) and after surgery (N = 6); five patients were foreigners (four Caucasian and one Black); seven patients had initial MRI but not CT scan data; 14 patients had received chemotherapy without operation at the hemato-oncology department; and six patients had coexisting cancers (one gastric, one pancreatic, two colon, one thyroid, and one gall bladder cancer). Therefore, 153 patients were included in this analysis (Fig. 2). Of these, 142 had ovarian cancers (91 serous, 20 mucinous, 14 clear cell, 12 endometrioid, 3 transitional, and 4 malignant mixed mesodermal tumors (MMMTs), 8 had primary peritoneal cancer, and 1 had fallopian tubal cancer. Nine cases of peritoneal and fallopian tubal cancers were serous. Patients underwent primary debulking surgery followed by adjuvant chemotherapy, which consisted of a minimum of six 21-day cycles of intravenous carboplatin (area under the curve 5) plus paclitaxel (175 mg/m^2^ body surface area). NAC was administered as in the adjuvant setting but only for two or three cycles, and interval debulking surgery followed after 3 or 4 weeks of rest. This study was reviewed and approved by the Institutional Review Board of Chung-Ang University Hospital (irb@caumc.or.kr; approval number: 2104-002-19360; date: April 29, 2021).

### Body composition analysis using CT images

**Fig. 3:**
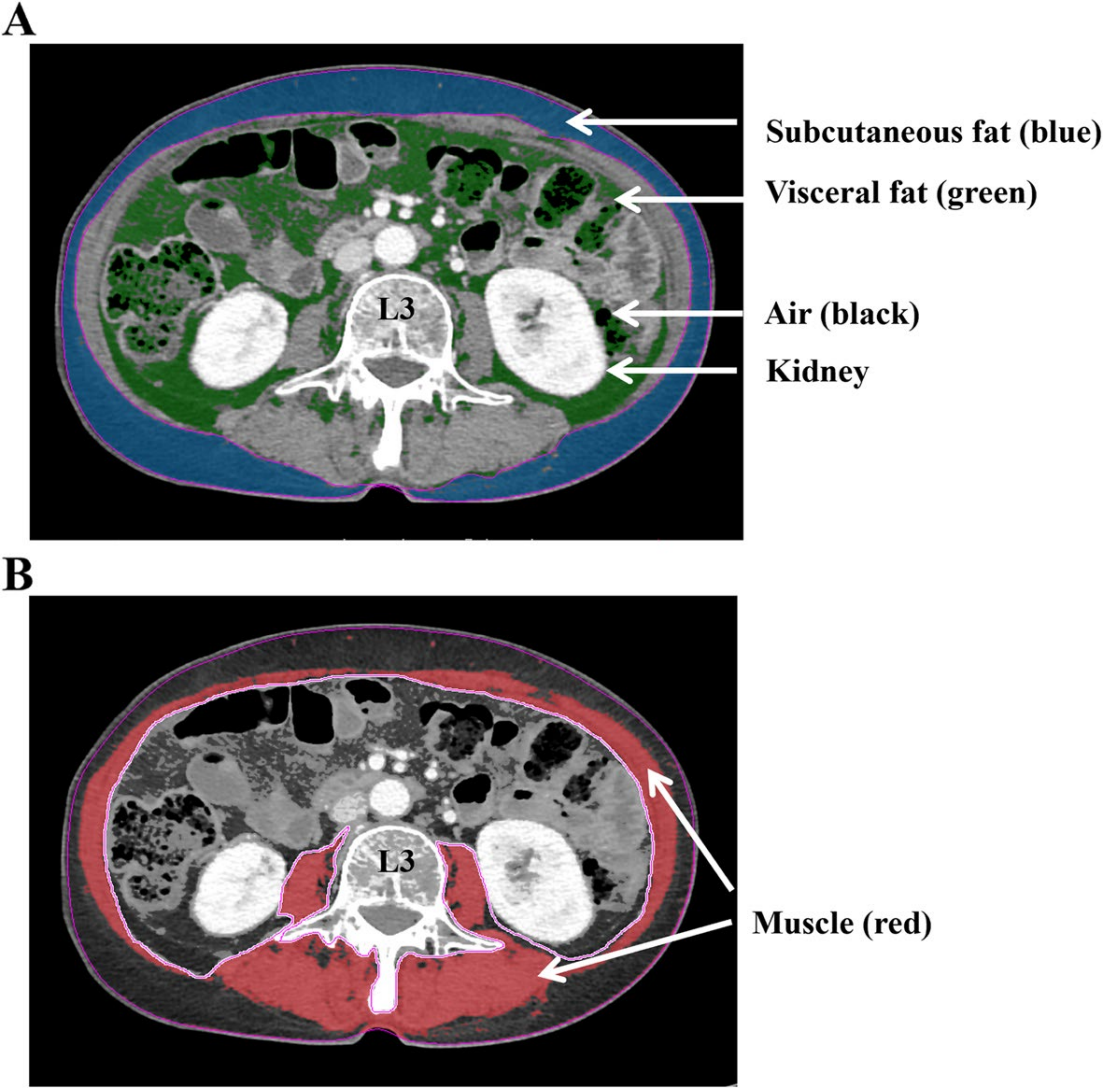
Schematic CT images at L3 level. Subcutaneous and visceral fat areas are marked in blue and green, respectively (**A**). Red represents skeletal muscle area (**B**)

For body composition analysis, fat area and SMA were measured using commercial imaging software (TeraRecon Aquarius; TeraRecon, USA). A single slice at the L3 level of the CT scan at diagnosis, with both transverse processes visible, was selected, and the areas were calculated automatically. According to predefined Hounsfield units (HU) [38], the software calculated the SMA (-29 to +150 HU), visceral fat area (-150 to -50 HU), and subcutaneous fat area (-190 to -30 HU) (Fig. 3A and B). In this standard method, the fat of bowel feces was inevitably included in visceral fat. Total fat area was calculated as the sum of the visceral and subcutaneous fat areas. Skeletal muscle attenuation was assessed by calculating the average HU value of the total muscle area. The fat-to-muscle ratio (FMR) was defined as the ratio of fat area to lean SMA. tFMR and vFMR were calculated using the total and visceral fat areas, respectively. In this study, the FMR value was used in clinical analysis because our preliminarily data showed that massive ascites influenced the fat area and muscle area but not the FMR (Fig. S1). SMI was calculated by dividing the SMA (cm^2^) by the square of the patient’s height (m^2^). Sarcopenia was defined as an SMI < 38.7 cm^2^/m^2^ according to the proposed cut-off value for patients with ovarian cancer [31].

### Data collection

Clinicopathological and laboratory data were collected via chart review. These included age, body weight, height, history of DM/HTN, menopausal status, histological type, serum cancer antigen (CA)-125 level, implementation of NAC, surgical optimality, FIGO stage, WBC count, neutrophil and lymphocyte counts, hemoglobin, platelet count, albumin, and PNI: 10x serum albumin (g/dL) + 0.005 × peripheral blood lymphocyte count (/μL). Comorbidities included in the NCI Comorbidity Index were screened [39]. One patient had stable angina, and three patients had asthma. These four patients had optimal conditions for surgery and underwent primary debulking surgery without complications. Due to the small number, we omitted comorbidity in the parameters. Surgical optimality was defined as a residual tumor < 1 cm after surgery. Chemoresistance was defined as recurrence within 6 months of first-line treatment.

### Statistical analysis

DFS was defined as the time from the last therapy to the diagnosis of the first recurrence, and OS was defined as the time in months from the last therapy to disease-related death. Survival was estimated using Kaplan–Meier estimates and compared with a log-rank test, where indicated. Multivariate analysis was performed using Cox regression analysis. Mean counts were analyzed using the Student’s *t*-test because the distributions of both populations were equal. Dichotomous groupings were analyzed using chi-squared and Fisher’s exact tests, as appropriate. All *p*-values reported were two-sided, and statistical significance was defined as *p* < 0.05. Statistical analysis was performed using the Statistical Package for the Social Sciences (SPSS, version 15.0, Chicago, IL, USA).

## Supplementary Information

**Additional file 1: Figure S1.** Comparison of fat and muscle areas in patients with massive ascites before and after two cycles of neoadjuvant chemotherapy. Visceral fat and smooth muscle areas in CT images at L3 level marked in green and red, respectively, before treatment (A and B) and after treatment (C and D). Each value is shown (E), and no significant difference in vFMR was noted.

## Data Availability

The datasets generated during the current study are available from the corresponding author on reasonable request.
