# Supplementary figures and images for: High visceral fat-to-muscle ratio is an independent factor that predicts worse overall survival in patients with primary epithelial ovarian, fallopian tube, and peritoneal cancer
Source: J Ovarian Res. 2023 Jan 21;16:19. doi: 10.1186/s13048-023-01098-1 (PMC9863081; doi:10.1186/s13048-023-01098-1)

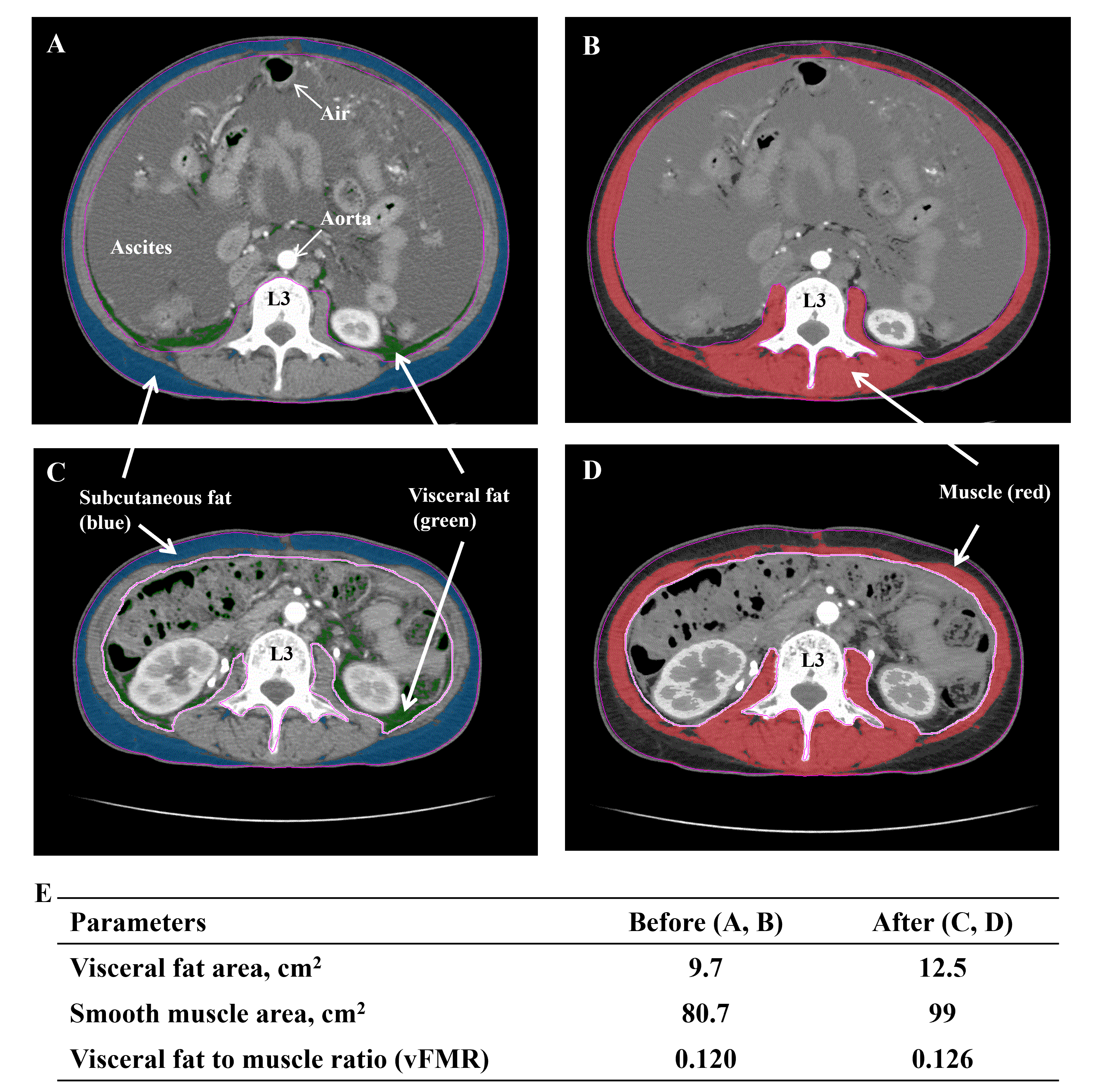

Supplement: Supplementary file 1 — Additional file 1: Figure S1. Comparison of fat and muscle areas in patients with massive ascites before and after two cycles of neoadjuvant chemotherapy. Visceral fat and smooth muscle areas in CT images at L3 level marked in green and red, respectively, before treatment (A and B) and after treatment (C and D). Each value is shown (E), and no significant difference in vFMR was noted. [file 13048_2023_1098_MOESM1_ESM.tif]
